# Marein Prevented LPS-Induced Osteoclastogenesis by Regulating the NF-&kappa;B Pathway In Vitro

**DOI:** 10.4014/jmb.2109.09033

**Published:** 2022-01-12

**Authors:** Yuling Li, Jing Zhang, Caiping Yan, Qian Chen, Chao Xiang, Qingyan Zhang, Xingkuan Wang, Ke Jiang

**Affiliations:** 1 Department of Orthopaedics, Affiliated Hospital of North Sichuan Medical College, No. 63 Wenhua Road, Nanchong City, Sichuan Province 637000, P.R. China; 2 Department of Gastroenterology, Affiliated Hospital of North Sichuan Medical College, No. 63 Wenhua Road, Nanchong City, Sichuan Province 637000, P.R. China

**Keywords:** Marein, LPS, inflammation, RAW264.7, NF-κB

## Abstract

Many bone diseases such as osteolysis, osteomyelitis, and septic arthritis are caused by gram-negative bacterial infection, and lipopolysaccharide (LPS), a bacterial product, plays an essential role in this process. Drugs that inhibit LPS-induced osteoclastogenesis are urgently needed to prevent bone destruction in infective bone diseases. Marein, a major bioactive compound of *Coreopsis tinctoria*, possesses anti-oxidative, anti-inflammatory, anti-hypertensive, anti-hyperlipidemic, and anti-diabetic effects. In this study, we measured the effect of marein on RAW264.7 cells by CCK-8 assay and used TRAP staining to determine osteoclastogenesis. The levels of osteoclast-related genes and NF-&kappa;B-related proteins were then analyzed by western blot, and the levels of pro-inflammatory cytokines were quantified by ELISA. Our results showed that marein inhibited LPS-induced osteoclast formation by osteoclast precursor RAW264.7 cells. The effect of marein was related to its inhibitory function on expressions of pro-inflammatory cytokines and osteoclast-related genes containing RANK, TRAF6, MMP-9, CK, and CAII. Additionally, marein leads to markedly inhibited NF-&kappa;B signaling pathway activation in LPS-induced RAW264.7 cells. Concurrently, when the NF-&kappa;B signaling pathway was inhibited, osteoclast formation and pro-inflammatory cytokine expression were decreased. Collectively, marein could inhibit LPS-induced osteoclast formation in RAW264.7 cells via regulating the NF-&kappa;B signaling pathway. Our data demonstrate that marein might be a potential drug for bacteria-induced bone destruction disease. Our findings provide new insights into LPS-induced bone disease.

## Introduction

Osteoclasts, which are differentiated from the monocyte-macrophage lineage, can decrease the density of bone [1]. They generally occur in abundance in the presence of diseases including osteopetrosis, osteoporosis, rheumatoid arthritis, inflammatory osteolysis, and pycnodysostosis [2]. As reported, macrophage colony-stimulating factor (M-CSF) and receptor-activated nuclear factor kappa-B (NF-κB) ligand (RANKL) are pivotal molecules for osteoclast differentiation [3-5]. RANKL binding to and activating RANK results in osteoclast differentiation. Thus, these elements are very crucial for bone remodeling as they can stimulate osteoclast differentiation and activation [6].

Lipopolysaccharide (LPS) is a core antigen of gram-negative bacteria, which activate the innate immune system of the host [7]. LPS is an endotoxin that provides a persistent inflammatory stimulus to the tissue [8]. Research has found that LPS could promote inflammatory bone loss, and it can also lead to osteolytic diseases such as osteomyelitis, septic arthritis, periodontitis, and infection of orthopedic implants [9]. Furthermore, production of tumor necrosis factor (TNF)-α, interleukin (IL)-1β, prostaglandin E2 (PGE2), and IL-6 is known to be induced by LPS. Together these factors enhance RANKL expression while also recruiting a large number of adaptor proteins such as TNF receptor-associated factor 6 (TRAF6) and upregulating the expression of osteoclast differentiation genes, ultimately leading to destructive bone loss [10-12]. Therefore, exploring the molecular mechanism behind LPS intervention in bone metabolism disorders is of great significance.

**Fig. 1:**
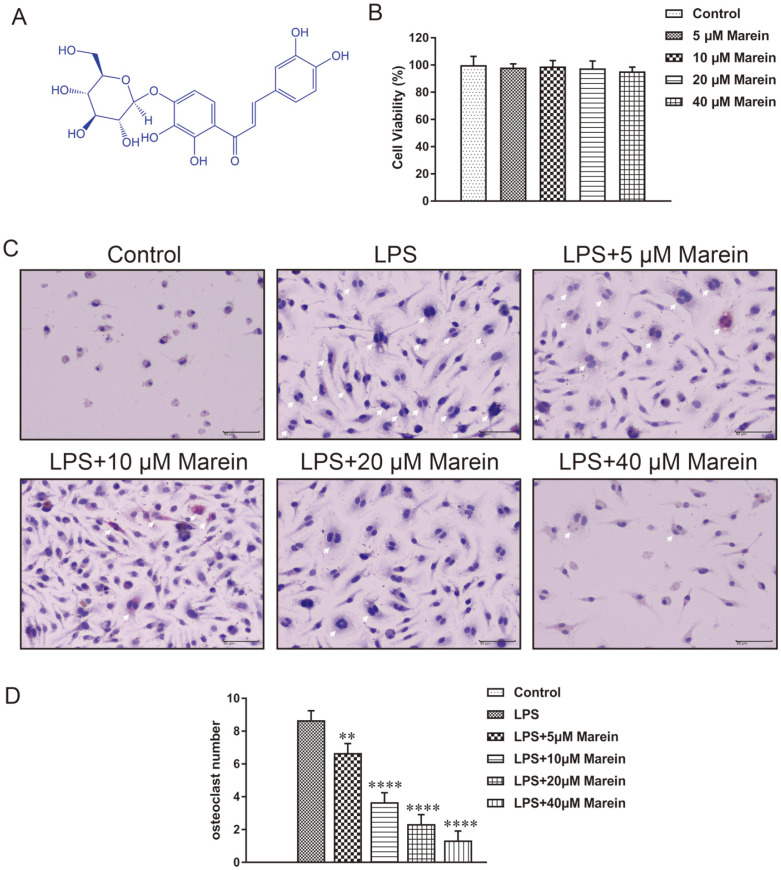
Marein inhibited LPS-induced osteoclastogenesis. (**A**) The chemical structure of marein. (**B**) RAW264.7 cells viability was measured by CCK-8 assay. (**C**) RAW264.7 cells were stained for TRAP staining and cells containing more than three nuclei were counted as osteoclasts under IX70 microscope. Bars = 10 μm. (**D**) The osteoclast number was counted per field of microscope and the number in the control group was zero. Bars represent the mean ± S.D. from three independent experiments. **p < 0.01 and ****p < 0.0001 compared with LPS-treated group.

Marein is a major bioactive compound of *Coreopsis tinctoria* [13, 14]. The chemical structure formula of marein is shown in Fig. 1A. As previous studies described, marein shows activities that are beneficial in bone diseases [15]. For instance, Yao, M *et al*. have reported that marein could inhibit the generation of ROS and the activation of the NF-kB pathway in vitro. This suggests that marein has anti-oxidative and anti-inflammatory activities [16]. Nevertheless, to the best of our knowledge, there are no studies regarding the function of marein on osteoclastogenesis. Considering the role of the NF-κB signaling pathway in osteoclast differentiation and the activities of marein [17], we aimed to determine the effects of marein on osteoclastogenesis in RAW264.7 cells based on the NF-κB signaling pathway.

## Materials and Methods

### RAW264.7 Cell Culture and Treatment

Osteoclast precursor RAW264.7 cells (China) were cultured in Dulbecco’s Modified Eagle’s Medium (DMEM) with 5% CO_2_ at 37°C, and supplemented with 10% fetal bovine serum, 100 units/ml penicillin, and 100 μg/ml streptomycin. This was followed by pretreatment for 1 h with marein (Wuhan ChemFaces Biochemical Co., Ltd., China) at 5, 10, 20, and 40 μM, followed by treatment with LPS (100 ng/ml) for 24 h, respectively. For inhibitor studies, the culture was treated with sulfasalazine (NF-κB pathway inhibitor, Med Chem Express) for 30 min, following the treatment with marein.

### Assessment of Cell Viability by Counting Kit-8 (CCK-8) Assay

CCK-8 was performed to detect whether marein had toxic effects on the RAW264.7 cells. RAW264.7 cells were cultured to the logarithmic growth phase, and the cell concentration was adjusted to 1 × 10^5^ ml/cell. For cell culturing, 96-well cell plates were used and each well contained 100 μl. After the cells adhered, marein was added into each well of the original medium at final concentrations of 0 μM, 5 μM, 10 μM, 20 μM, and 40 μM. After culturing overnight, 10 μl CCK-8 solution was added per well, then cultured 4 h, and the absorbance of each well was measured at 450 nm. Samples were done in quadruplicate.

### In Vitro Osteoclastogenesis Assay

The effects of marein on LPS-induced osteoclast were assessed, and 96-well plates were used to culture RAW264.7 cells at approximately, 1 × 10^3^ cells/well. Cells were treated with or without 100 ng/ml LPS alone or in combination with 5, 10, 20, and 40 μM marein, respectively. Groups were as follows: Control, LPS, LPS+5 μM marein, LPS+10 μM marein, LPS+20 μM marein and LPS+40 μM marein. Other groups were as follows: Control, LPS, LPS+40 μM marein, and LPS+sulfasalazine (NF-κB inhibitor). Then, cells were washed twice before fixing the cells and staining for TRAP. Finally, the TRAP-positive multinucleated cells (MNCs) were counted by IX70 microscope (Olympus, Japan).

### Western Blotting

After the marein and LPS treatment of RAW264.7 cells, RANK, TRAF6, matrix metalloproteinase 9 (MMP-9), cathepsin K (CK), carbonic anhydrase II (CAII), and inhibitor of nuclear factor kappa B α (IκBα) or phosphorylation-IκBα (p-IκBα) were detected by western blot. β-actin was used as a control. Sodium dodecyl sulfate-polyacrylamide gel electrophoresis (SDS-PAGE) was performed to separate proteins. Then, the cells were transferred onto polyvinyl difluoride (PVDF) membranes and the blots were blocked. After that, probing was done with rabbit anti-mouse antibodies at 4°C before culturing overnight and washing thrice. Subsequently, the blots were incubated for 2 h with a goat horseradish peroxidase-conjugated secondary antibody at r.t, washed for 10 min, and detected by chemiluminescence detection system. The bands were quantified by ImageJ software.

### Enzyme-Linked Immunosorbent Assay (ELISA)

Similarly, RAW264.7 cells were treated with different concentrations of marein and LPS. After that, TNF-α, IL-1β, PGE2, and cyclooxygenase-2(COX-2) antibodies were used to coat ELISA plates at 4°C overnight, followed by blocking the plates by BSA, before adding samples and incubating for 2 h at 37°C and washing. Then, horseradish peroxidase (HRP)-conjugated TNF-α, IL-1β, PGE2, and COX-2 antibodies were added and interacted for 1 h at 37°C before washing again and stopping the action to interact with the substrate for 10 min. Finally, OD was measured at λ= 450 nm.

### Statistical Analysis

Results are expressed as the mean ± SD. Statistical comparisons between groups were carried out by one-way ANOVA with post hoc tests to compare differences between experimental and control groups. Statistical analyses were performed using GraphPad Prism 7 software. Statistical significance was defined at *p* < 0.05. All the experiments were performed at least three times.

## Results

### Marein Inhibited LPS-Induced Osteoclast Formation

CCK-8 assay was performed to eliminate the inhibitory effects of marein on osteoclast formation in RAW264.7 cells. The data showed that the viability of the RAW264.7 cells was not distinctly damaged by marein, when the marein concentration was at 5, 10, 20, and 40 μM, indicating that marein is nontoxic to RAW264.7 cells under 5-40 μM (Fig. 1B).

Next, to observe the effect of marein on LPS-induced osteoclastogenesis, marein and LPS treatment and TRAP staining have shown that LPS could exactly stimulate the differentiation of RAW264.7 cells into osteoclasts. On the contrary, marein reversed this phenomenon, represented less round osteoclast and fewer nuclei (Fig. 1C). Marein showed a dose-dependent manner for inhibition of LPS-induced osteoclast formation (Fig. 1D). Notably, 40 μM marein showed the most inhibition of osteoclast than the other three groups. The average quantity of osteoclast was only 12.5% of the LPS-treated group, while at least three different microscope fields were counted. Moreover, 40 μM marein displayed the most inhibitory activity against osteoclast in three marein treatment groups.

### Marein Restrained LPS-Induced Osteoclast-Related Gene Expression

**Fig. 2:**
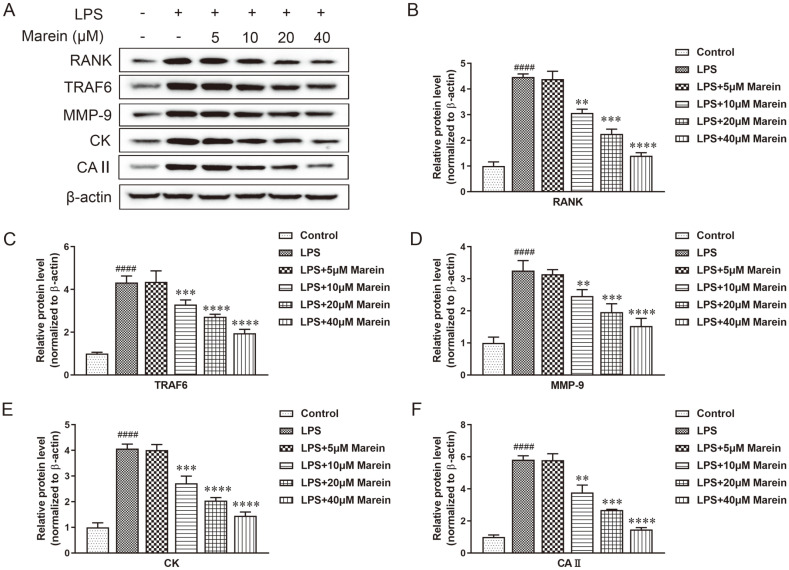
Marein restrained LPS-induced osteoclast-related protein expression. Respectively, 5, 10, 20 and 40 μM marein was used to treated RAW264.7 cells with or without LPS for 24h. (**A**) Osteoclast-related proteins were analyzed by western blotting, and these included RANK, TRAF6, MMP-9, CK, and CAII. The relative density of MMP-9 (**B**), CK (**C**), RANK (**D**), TRAF6 (**E**), and CAII (F) were quantified and normalized to control. Bars represent the mean ± S.D. from three independent experiments. ^####^ *p* < 0.0001 compared with the control group, **p < 0.01, ***p < 0.001, and ****p < 0.0001 compared with the LPS-treated group.

Western blotting (WB) was used to quantify expressions containing RANK, TRAF6, MMP-9, CK, and CAII, all of which are related to osteoclast differentiation. The WB bands containing all the former proteins were shown in Fig. 2A. Particularly, the data show that 100 ng/ml LPS observably enhanced proteins expression of RANK (Fig. 2B), TRAF6 (Fig. 2C), MMP-9 (Fig. 2D), CK (Fig. 2E), and CAII (Fig. 2F), while the formation of osteoclast was promoted by LPS. Marein treatment significantly suppressed the expression of the above proteins in a dose-dependent manner. Particularly, 40 μM marein exhibited the suppression on above proteins stronger than that of marein at 5, 10, and 20 μM.

### Marein Inhibited the Release of the Pro-Inflammatory Cytokines

**Fig. 3:**
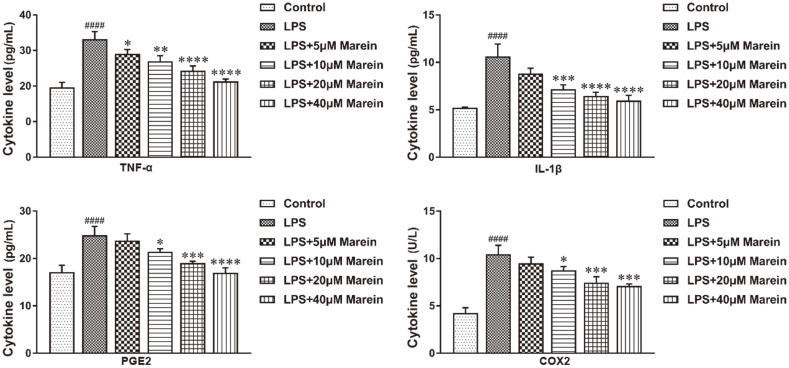
Marein suppressed the release of pro-inflammatory cytokines. The expressions of pro-inflammatory cytokines including TNF-α (**A**), PGE2 (**B**), IL-β (**C**), and COX-2 (**D**) were measured by ELISA. All values were given as means ± S.D. from three independent experiments. ^####^ *p* < 0.0001 compared with the control group, *p < 0.05, **p < 0.01, ***p < 0.001, and ****p < 0.0001 compared with the LPS-treated group.

Pro-inflammatory cytokines are considered to be negative mediators of bone loss [18]. Crucially, they resulted in obvious increases in TNF-α, IL-1β, PGE2, and COX-2 (Fig. 3), and their levels were increased compared with the control group, respectively. Clearly, marein treatment for 24 h suppressed LPS-induced secretion of these cytokines in a dose-dependent manner. Importantly, marein at 40 μM caused a more obvious reduction of TNF-α, IL-β, PGE2, and COX-2 compared with the remaining concentrations (Fig. 3).

### Marein Inhibited LPS-Triggered Activation of NF-κB Pathway

**Fig. 4:**
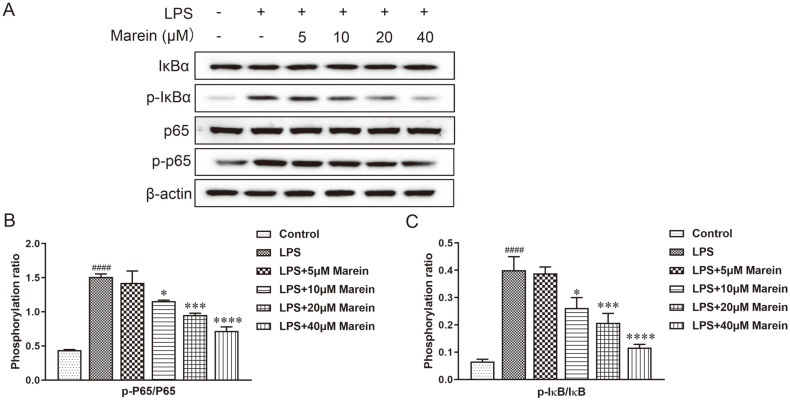
Marein inhibited LPS-triggered activation of NF-κB pathway in RAW264.7 cells. (**A**) The expression levels of IκBα and p-IκBα. (**B**) The relative level of p-IκBα/IκBα. All values are given as means ± S.D. from three independent experiments. ^####^ *p* < 0.0001 compared with the control group. *p < 0.05, ***p < 0.001, and ****p < 0.0001 compared with the LPStreated group.

The above results demonstrated that marein could suppress LPS-induced osteoclast formation as well as the expression of osteoclast-related genes. Therefore, to illuminate the potential molecular mechanisms of the suppression effect of marein on LPS-induced osteoclast formation, the change of the NF-κB pathway was explored. Given this, the ratio of p-IκBα/IκBα was analyzed by WB as bands (Fig. 4A). Results showed that the p-IκBα/IκBα ratio was upregulated by LPS in RAW264.7 cells, which presented the activation of the NF-κB pathway (Fig. 4B). Meanwhile, marein treatment efficiently inhibited LPS-increased NF-κB-related protein levels as assessed by western blot assay. Notably, the p-IκBα/IκBα ratio was decreased in a marein dose-dependent manner. Taken together, we illustrated that marein inhibited LPS-induced osteoclastogenesis by suppressing the activation of the NF-κB pathway.

### Inhibition of NF-κB Pathway Restrained LPS-Induced Osteoclast Formation and Inflammation

**Fig. 5:**
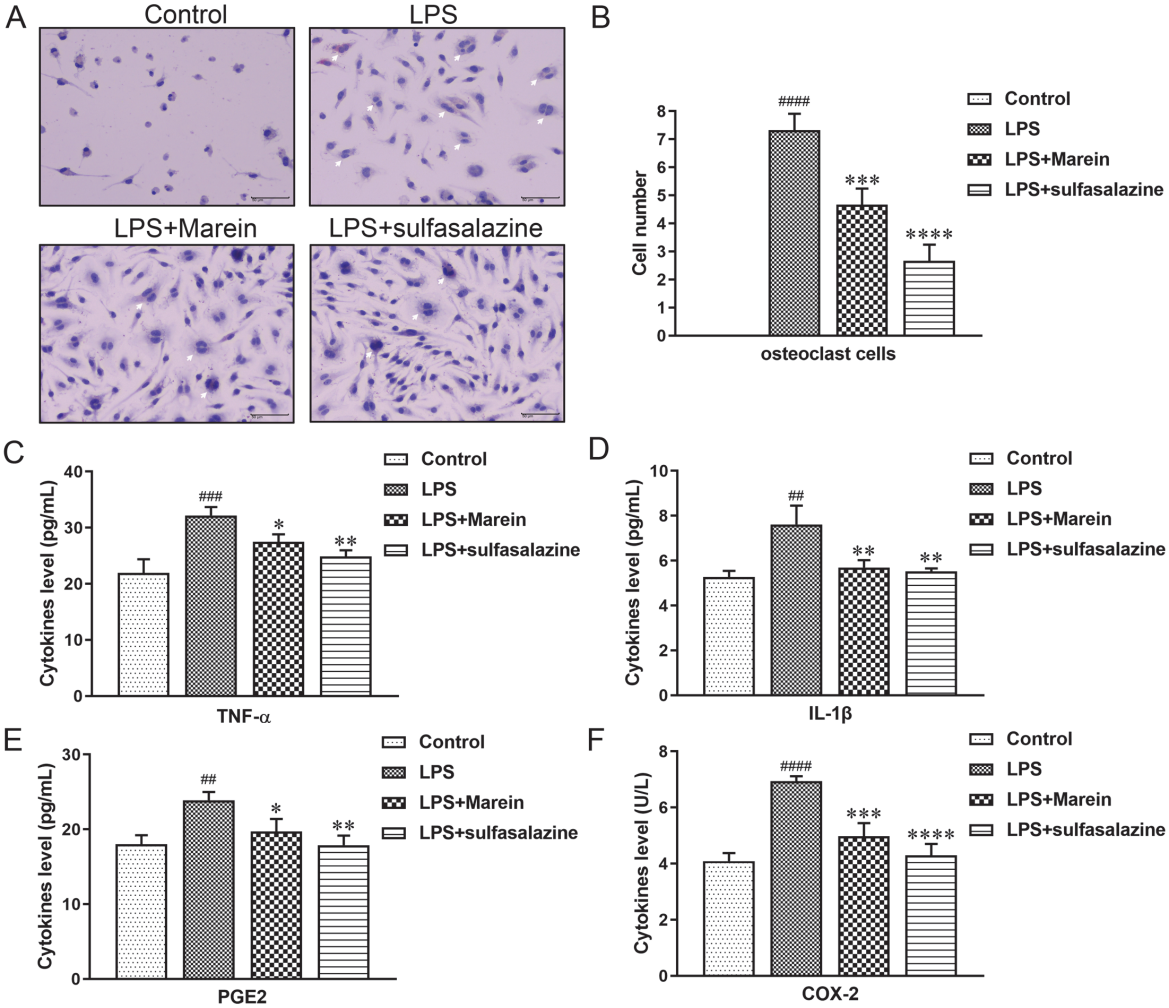
Restrained LPS-induced osteoclast formation and inflammation by inhibition of NF-κB pathway. (**A**) RAW264.7 cells were stained for TRAP staining and cells containing more than three nuclei were counted as osteoclast under the IX70 microscope. Bars = 10 μm. (**B**) The numbers of osteoclasts were counted per field of microscope and when the control group was zero. The expressions of pro-inflammatory cytokines TNF-α (**C**), IL-1β (**D**), PGE2 (**E**), and COX-2 (F) were measured by ELISA. All values are given as means ± S.D. from three independent experiments. ^##^ *p* < 0.01, ^###^ *p* < 0.001, and ^####^ *p* < 0.0001 compared with the control group. *p<0.05, **p<0.01, ***p<0.001, and ****p<0.0001 compared with the LPS-treated group. Sulfasalazine served as a positive control.

Next, the effects of inhibition of the NF-κB pathway on LPS-induced osteoclast formation and inflammation need to be elucidated. In the TRAP-staining assay, RAW264.7 cells were treated with 40 μM marein or sulfasalazine (NF-κB inhibitor) in the presence of LPS. Marein and sulfasalazine treatment caused osteoclasts to be less rounded with fewer nuclei than that of the LPS-treated group and indeed inhibited LPS-induced osteoclast formation (Fig. 5A). Notably, 40 μM marein could inhibit LPS-induced osteoclast formation, and the number of osteoclasts was 64% of the LPS-treated group. Moreover, osteoclastogenesis was inhibited in the sulfasalazine group compared with the LPS-treated group (Fig. 5B). Next, the contents of TNF-α, IL-1β, PGE2, and COX-2 in the culture supernatants were measured by ELISA. RAW264.7 cells were exposed to LPS and incubated with 40 μM marein or sulfasalazine for 24 h. LPS leads to the release of these pro-inflammatory cytokines. However, the expressions of LPS-induced TNF-α (Fig. 5C), IL-1β (Fig. 5D), PGE2 (Fig. 5E), and COX-2 (Fig. 5F) represented a downward trend, when treated with 40 μM marein and sulfasalazine. It indicated that suppression of the NF-κB pathway indeed decreased osteoclast formation and inflammation.

## Discussion

Persistent inflammatory responses could be caused by infectious bone diseases. At the same time, the affected sites may be secondary to local metabolic disorders, destruction, and death of bone [19, 20]. Considering the function of LPS and marein in modulating the immune system, it is worth studying the influence of marein on LPS-induced inflammation and osteoclasts. According to the results, we found that marein could inhibit LPS-induced osteoclastogenesis by suppressing NF-κB-mediated inflammatory response in RAW264.7 cells.

Related research confirmed that LPS could stimulate secretion of prostaglandins (PG), histamine, and other inflammatory mediators, and activate the release of immune factors such as interleukin (IL), TNF-α, and these inflammatory mediators can be produced by LPS stimulation through different mechanisms to promote the formation and bone resorption of osteoclast activity. Studies have demonstrated that LPS can promote osteoclast differentiation by enhancing COX-2 expression and activating RANK, JNK, and ERK1/2 pathways [21]. Furthermore, it has been reported that osteoclast differentiation also was accelerated by LPS via activating the NF-κB-NFATc1 signal pathway [22]. In this study, we found that the expressions of TRAP-positive cells, osteoclasts-related genes, and pro-inflammatory cytokines were all increased by LPS in RAW264.7 cells. However, these events were inhibited by marein, which indicated that marein could be a potential candidate for treating and/or preventing osteoclast-associated diseases, including osteoporosis.

Except for marein, some plant-derived natural organic compounds, such as isoflavones (coumestrol, daidzein, and genistein), have been shown in several studies to have a direct inhibitory effect on cytokine-induced osteoclast differentiation [23, 24]. In addition, another study illustrated that puerarin effectively inhibited the production of the inflammatory mediators (TNF-α, IL-1β, PGE2) induced by LPS [25]. It was also reported that artesunate reduced TNF-α production and prevented LPS-induced bone loss in vivo [1]. When suppressing the RANKL-induced activator protein-1 and NFATc1 signaling pathways, the differentiation of bone marrow macrophages into mature osteoclasts was inhibited [26]. Punicalagin also attenuated the LPS-induced inflammatory responses via downregulation of the FoxO3a/autophagy signaling pathway in RAW264.7 cells [27]. Following their results, we found that treatment with marein effectively inhibited the production of TNF-α, IL-1β, PGE2, and COX-2 induced by LPS, which were consistent with the inhibitory effect of marein on osteoclast formation. Therefore, marein could inhibit the production of these pro-inflammatory cytokines which are LPS-induced, and prevent later stages of osteoclast differentiation in infective bone destruction.

NF-κB was reported as the key transcription factor associated with the regulation of COX-2 and mPGES1 after LPS stimulation [28]. Abnormal activation of NF-κB signaling induced osteoclast formation by increasing expression of NFATc1 [29]. Islam *et al*. reported that LPS could induce osteoclastic cell differentiation in RAW264.7 cells as a potent bone-resorbing factor [30]. However, one report speculated about the molecular mechanisms underlying the LPS-induced RAW264.7-osteoclasts formation saying that LPS might mimic RANKL‐induced osteoclast formation via activation of NF-κB and SAPK/JNK [31]. Previous studies [32, 33] have indicated that the NF-κB pathway plays a central role in the regulation of osteoclast differentiation and survival. Therefore, the NF-κB pathway could serve as a therapeutic target in the inhibition of osteoclastogenesis.

Importantly, we found that marein treatment markedly decreased the osteoclast numbers and downregulated the expression of pro-inflammatory cytokines through inhibiting LPS-induced activation of NF-κB, which has critical meaning for osteoclast differentiation. A primary level of control for NF-κB was through interactions with an inhibitor protein called IκB which retained NF-κB in the cytoplasm through masking of the nuclear localization sequences. Removal of IκB activated the NF-κB NLSs so that NF-κB rapidly translocated into the nucleus, bound to select gene promoters in a sequence-specific manner, and activated gene transcription [34]. Activation of NF-κB to move into the nucleus was controlled by the targeted phosphorylation and subsequent degradation of IκBα. Therefore, the upregulated ratio of p-IκBα/IκBα could present the activation of the NF-κB pathway [35]. In this study, we found that marein could inhibit the NF-κB pathway by decreasing the ratio of p-IκBα/IκBα and downregulate the levels of pro-inflammatory cytokines including TNF-α, IL-1β, PGE2, and COX-2. Encouragingly, the inhibitory effect of marein was similar to that of sulfasalazine. In LPS-treated RAW264.7 cells, these results indicated that marein could inhibit NF-κB activation by suppressing the phosphorylation of IκBα. Considering the key role of the NF-κB signaling pathway in LPS-induced osteoclast formation [32, 33], we concluded that suppression of NF-κB might be a target for inhibitory osteoclast formation, and marein may serve as an effective therapeutic agent to treat bone loss diseases.

Our findings introduced a novel therapeutic drug to control bacteria-induced bone destruction disease and demonstrated the important role of the NF-κB signaling pathway in this regulating process. Moreover, marein exhibited inhibitory effects on LPS-induced osteoclastogenesis by inhibiting the NF-κB signaling pathway in vitro. Our research may provide a reference for correct evaluation of the pharmacological effect of these natural plant drugs on bone tissue in an inflammatory state as well as a direction for the application of such drugs in the regulation of bone metabolism in infectious bone disease. Nevertheless, the effect of marein on LPS-induced bone loss in vivo still needs to be proved.
